# Pexidartinib and Immune Checkpoint Inhibitors Combine to Activate Tumor Immunity in a Murine Colorectal Cancer Model by Depleting M2 Macrophages Differentiated by Cancer-Associated Fibroblasts

**DOI:** 10.3390/ijms25137001

**Published:** 2024-06-26

**Authors:** Daisuke Shimizu, Ryo Yuge, Yuki Kitadai, Misa Ariyoshi, Ryo Miyamoto, Yuichi Hiyama, Hidehiko Takigawa, Yuji Urabe, Shiro Oka

**Affiliations:** Department of Gastroenterology, Hiroshima University Hospital, Hiroshima 734-0037, Japan; dshimizu@hiroshima-u.ac.jp (D.S.); s025eb@hiroshima-u.ac.jp (Y.K.); misa4235@hiroshima-u.ac.jp (M.A.); ryo4book@hiroshima-u.ac.jp (R.M.); yhiyama@hiroshima-u.ac.jp (Y.H.); hidehiko@hiroshima-u.ac.jp (H.T.); beyan13@hiroshima-u.ac.jp (Y.U.); oka4683@hiroshima-u.ac.jp (S.O.)

**Keywords:** pexidartinib, CSF-1R inhibitor, colorectal cancer, cancer-associated fibroblasts, tumor-associated macrophages

## Abstract

Tumor-associated macrophages (TAMs) and cancer-associated fibroblasts (CAFs) are known to play supportive roles in tumor development and progression, but their interactions in colorectal cancer (CRC) remain unclear. Here, we investigated the effects of colon-cancer-derived CAFs on TAM differentiation, migration, and tumor immunity, both in vitro and in vivo. When co-cultured with monocytes, CAFs attracted monocytes and induced their differentiation into M2 macrophages. Immunohistology of surgically resected human CRC specimens and orthotopically transplanted mouse tumors revealed a correlation between numbers of CAFs and numbers of M2 macrophages. In a mouse model of CRC orthotopic transplantation, treatment with an inhibitor of the colony-stimulating factor-1 receptor (PLX3397) depleted M2 macrophages and increased CD8-positive T cells infiltrating the tumor nest. While this treatment had a minor effect on tumor growth, combining PLX3397 with anti-PD-1 antibody significantly reduced tumor growth. RNA-seq following combination therapy showed activation of tumor immunity. In summary, CAFs are involved in the induction and mobilization of M2 macrophage differentiation in the CRC tumor immune microenvironment, and the combination of cancer immunotherapy and PLX3397 may represent a novel therapeutic option for CRC.

## 1. Introduction

Colorectal cancer (CRC) has high morbidity and mortality, with especially poor prognosis in advanced stages [1]. While immunotherapy has recently been reported to have high efficacy against multiple cancer types, it has limited efficacy against CRC [2,3]; with mechanisms influencing the immune response remaining to be elucidated. In CRC, there is an urgent need to identify concomitant drugs that enhance the efficacy of cancer immunotherapy.

CRC has been reported to be resistant to immune checkpoint inhibitor monotherapy due to the exclusion of CD8-positive T cells, required to activate antitumor immunity, from the tumor microenvironment [4]. We previously reported that in CRC with abundant cancer-associated fibroblasts (CAFs), CD8-positive T cells are trapped in the tumor stroma and unable to infiltrate into the tumor nest [5], suggesting that among the multiple cell types recruited by and chemokines produced by CAFs, some or all inhibit CD8-positive T cell infiltration.

Multiple studies have shown that CAFs regulate immune cell mobilization and function [6,7]. They promote monocyte recruitment to tumors and promote differentiation into tumor-associated macrophages (TAMs) in CRC, oral squamous cell carcinoma, and breast cancer [8,9,10,11]. TAMs present in the tumor microenvironment significantly influence its formation, including the extracellular matrix, tumor metabolism, and angiogenesis, stimulating tumor growth [12]. TAMs are primarily classified as M1 or M2 macrophages [13], with M1 involved in biological defense, such as antitumor activity, and M2 involved in tumor growth. M2 macrophages are primarily localized in the tumor microenvironment in advanced CRC [14,15,16]. Finally, M2 macrophages are also reported to suppress tumor immunity in several cancer types [17].

Even though TAMs and CAFs are crucial components of the tumor stroma, their interactions in CRC remain poorly understood. To our knowledge, no studies have examined the role of CRC CAFs in the induction of monocyte migration and differentiation into macrophages, specifically M2 macrophages, in vitro or in vivo, including in human clinical specimens. Based on the findings reported to date, we hypothesized that in CRC, CAFs also enhance monocyte mobilization into the tumor and promote their differentiation into TAMs (predominantly M2 macrophages); we also hypothesized that M2 macrophages suppress infiltration of CD8-positive T cells into the tumor.

Monocytes in the peripheral blood are thought to migrate to tissues and differentiate into TAMs under the influence of humoral factors produced by tumor and stromal cells. Colony-stimulating factor-1 (CSF-1) is recognized as one of the major factors involved in this process [18,19,20]. The CSF-1 receptor (CSF-1R) is expressed on the surfaces of monocytes and TAMs; it has been reported that treatment with the CSF-1R inhibitor PLX3397 (Pexidartinib) depletes TAMs in tumors, including in mouse models of breast cancer and malignant peripheral nerve sheath tumors [21,22].

One of the objectives of this study was to investigate the effects of CAFs on M2 macrophage differentiation and migration in CRC, using both CRC cell lines and human CRC specimens. The second objective was to evaluate the effect of PLX3397 on the tumor immune microenvironment and its efficacy when combined with cancer immunotherapy using a murine CRC orthotopic transplant model.

## 2. Results

### 2.1. Monocyte Differentiation into M2 Macrophages Induced by CAFs Derived from CRC Cells

**Figure 1:**
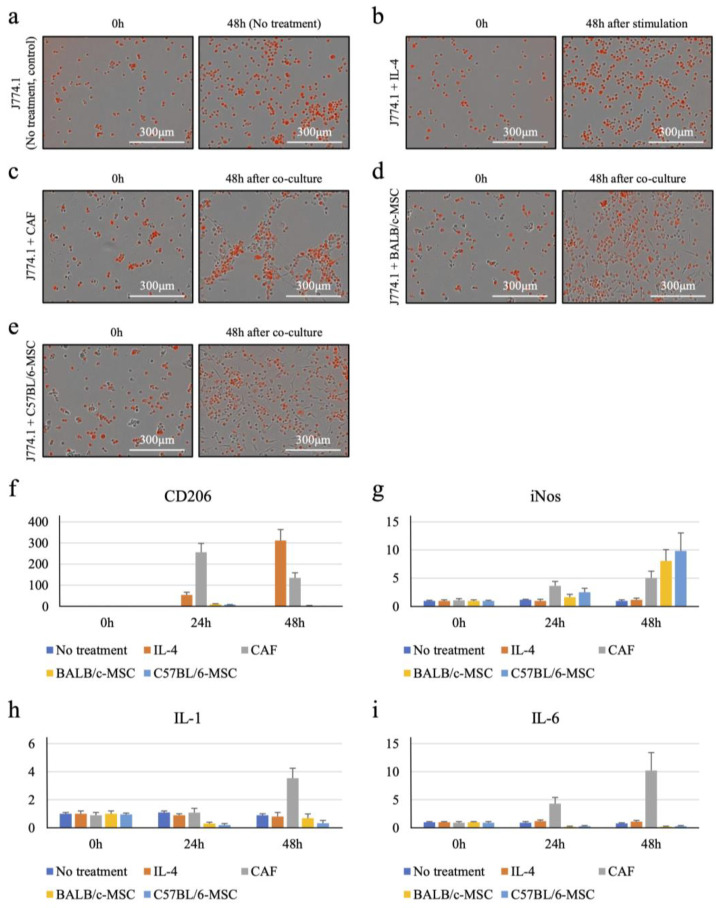
In culture, cancer-associated fibroblasts (CAFs) derived from colorectal cancer (CRC) cells attract J774.1 cells (transfected with a construct expressing fluorescent luciferase) to the CAF periphery and induce their differentiation into M2 macrophages. (**a**) J774.1 cells in monoculture incubated for 48 h. (**b**) J774.1 cells in monoculture incubated with IL-4 for 48 h. (**c**) J774.1 cells and CAFs co-cultured for 48 h. (**d**) J774.1 cells co-cultured with BALB/c MSCs for 48 h. (**e**) J774.1 cells co-cultured with C57BL/6 MSCs for 48 h. (**f**–**i**) Expression measured using quantitative reverse transcription-polymerase chain reaction (qRT-PCR) in J774.1 cells cultured alone, cultured with IL-4, and co-cultured with CAFs, BALB/c MSCs, and C57BL/6 MSCs for 48 h, of (**f**) the M2 marker CD206; (**g**) the M1 marker iNos; (**h**) the M1 marker IL-1; and (**i**) the M1 marker IL-6. Data are presented as means ± s.d.

The J774.1 cell line was cultured alone as a control (Figure 1a) concurrently with cultures that were stimulated by IL-4 for 48 h. IL-4 stimulation did not alter J774.1 morphology over time (Figure 1b). Using quantitative reverse transcription-polymerase chain reaction (qRT-PCR), expression of the M2 marker CD206 increased 55.4-fold and 312-fold at 24 h and 48 h of stimulation, respectively (Figure 1f). Expression of the M1 macrophage markers iNos, IL-1, and IL-6 was not detected (Figure 1g,h,i, respectively). These results confirm that J774.1 cells differentiate into M2 macrophages upon IL-4 stimulation.

We co-cultured J774.1 cells with murine CAFs. Micrographs acquired after 48 h of co-culture suggested that J774.1 cells were more abundant around CAFs in some areas (Figure 1c). After co-culture, J774.1 cells were analyzed using flow cytometry, and expression levels of each marker were measured by qRT-PCR. Expression of the M2 macrophage marker CD206 increased 256-fold at 24 h and 135-fold at 48 h (Figure 1f). Expression of the M1 macrophage markers iNos, IL-1, and IL-6 was either not detected or detected only in trace amounts (Figure 1g,h,i, respectively). These results suggest that CAFs recruit monocytes and induce the differentiation of monocytes into M2 macrophages.

Mesenchymal stem cells (MSCs) are the precursors of CAFs, and they are generally thought to only function as stem cells and are not thought to behave like CAFs. To test this, we co-cultured J774.1 cells with MSCs from BALB/c or C57BL/6 mice. Unlike the CAF co-cultures, the J774.1 cells were scattered instead of being concentrated around the MSCs at 48 h (Figure 1d,e). After co-culture, J774.1 cells were analyzed for marker expression levels using flow cytometry and qRT-PCR. The two MSC lines increased expression of the M2 marker CD206 very slightly, but much less than CAFs did (Figure 1f). The M1 markers iNos, IL-1, and IL-6 were either not detected or detected at low levels (Figure 1g,h,i, respectively). The expression level of CD206 in J774.1 cells was highest after 24 h of co-culture with CAFs, followed by IL-4 stimulation; it was lowest when co-culturing with MSCs (Figure 1f).

### 2.2. CAF Abundance Correlates with the Abundance of M2 Macrophages in the Human CRC Microenvironment

**Figure 2:**
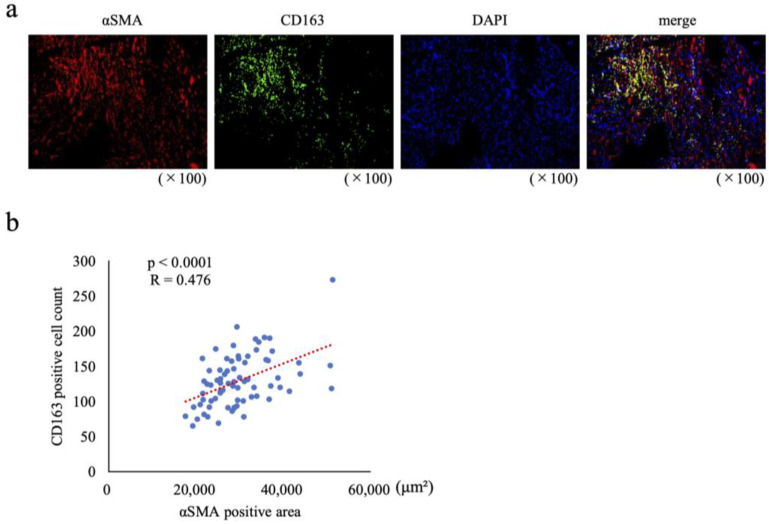
Abundance of cancer-associated fibroblasts (CAFs) correlates with abundance of M2 macrophages in human colorectal cancer (CRC). (**a**) Immunofluorescence staining for αSMA and CD163. (**b**) Scatterplot from 73 samples of αSMA-positive areas and CD163-positive cell counts. R, correlation coefficient.

Fluorescence immunostaining for the CAF marker αSMA and the M2 macrophage marker CD163 in 73 clinical CRC tumor samples allowed for quantification of αSMA-positive areas and abundance of CD163 positive cells in the tumor area (Figure 2a). Clinical characteristics of the 73 samples are shown in Table 1. Scatter plots and regression analysis showed a moderately strong correlation between CAF abundance and M2 macrophage abundance with a correlation coefficient (R) of 0.476 and *p* < 0.0001 (Figure 2b).

### 2.3. CAFs Increase M2 Macrophage Abundance in an Orthotopic Transplant Mouse Model of CRC

**Figure 3:**
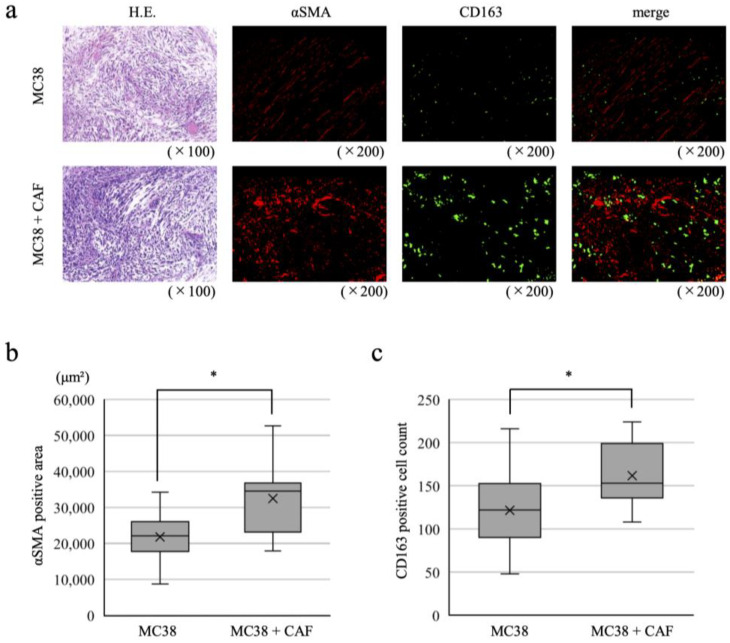
Cancer-associated fibroblasts (CAFs) increase M2 macrophage abundance in an orthotopic transplant mouse model of colorectal cancer (CRC). (**a**) Fluorescence immunostaining for αSMA and CD163 in transplanted tumors in a CRC orthotopic mouse model with MC38-only control (top row) and MC38 and CAFs co-transplanted (bottom row) into C57BL/6 mice. (**b**) αSMA-positive areas. (**c**) Numbers of CD163-positive cells. Data are presented as means ± s.d.; * *p* < 0.01 by unpaired two-sided Student’s *t*-test.

**Figure 4:**
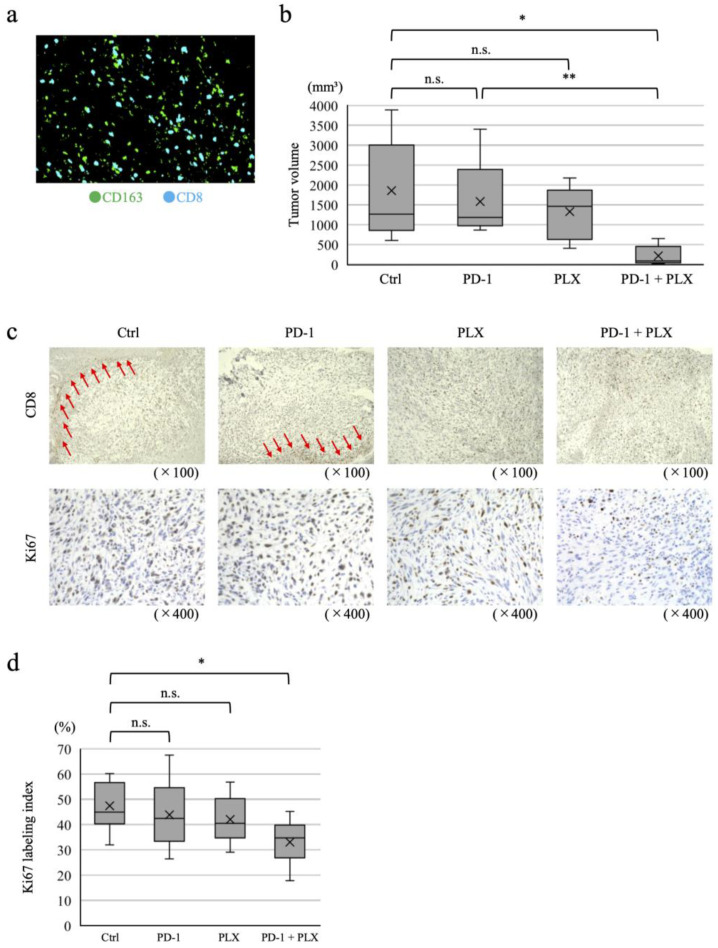
M2 macrophages inhibit tumor immunity by blocking tumor infiltration of CD8-positive T cells. (**a**) CD8-positive T cells in contact with M2 macrophages in tumor stromal-rich areas observed by fluorescence immunostaining for CD163 and CD8 in a colorectal cancer (CRC) orthotopic mouse model using co-transplantation of MC38 and cancer-associated fibroblasts (CAFs). (**b**) Tumor volumes 35 days after treatment in control, anti-PD-1 antibody monotherapy, PLX3397 monotherapy, and combination therapy groups, using the orthotopic transplant mouse model with co-transplantation. (**c**) Immunostaining for CD8 and Ki67. Red arrows indicate CD8-positive cells accumulating in the stroma around the tumor nest. (**d**) Ki67 labeling indices. Data are presented as means ± s.d.; * *p* < 0.01; ** *p* < 0.05; n.s., not significant using an unpaired, two-sided Student’s *t*-test.

Two mouse models were created, one in which the only the mouse CRC cell line MC38 was transplanted into the cecum of mice and the other in which CAFs and MC38 were co-transplanted. The resulting tumors were analyzed five weeks after transplantation. Fluorescence immunostaining for the CAF marker αSMA and the M2 macrophage marker CD163 was performed for all resected tumors to quantify αSMA-positive areas and numbers of CD163-positive cells in the tumor area (Figure 3a). With co-transplantation, αSMA-positive areas were significantly increased compared to the MC38-only controls, accompanied by a significant increase in the abundance of CD163-positive cells (Figure 3b,c). Similarly, in these murine models, it is possible that CAFs recruit monocytes to the tumor site and induce differentiation of monocytes into M2 macrophages. Finally, fluorescence immunostaining for CD8 revealed many CD8-positive T cells in contact with M2 macrophages in the stromal-rich areas (Figure 4a).

### 2.4. Combination Therapy with PLX3397 and Anti-PD-1 Antibody Further Promotes CD8-Positive T Cell Infiltration at the Tumor Site and Reduces Tumor Volume

**Figure 5:**
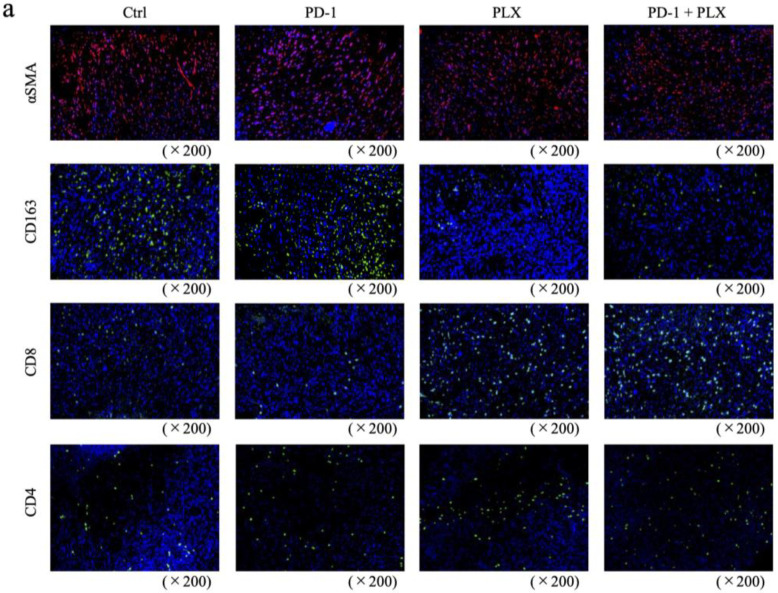

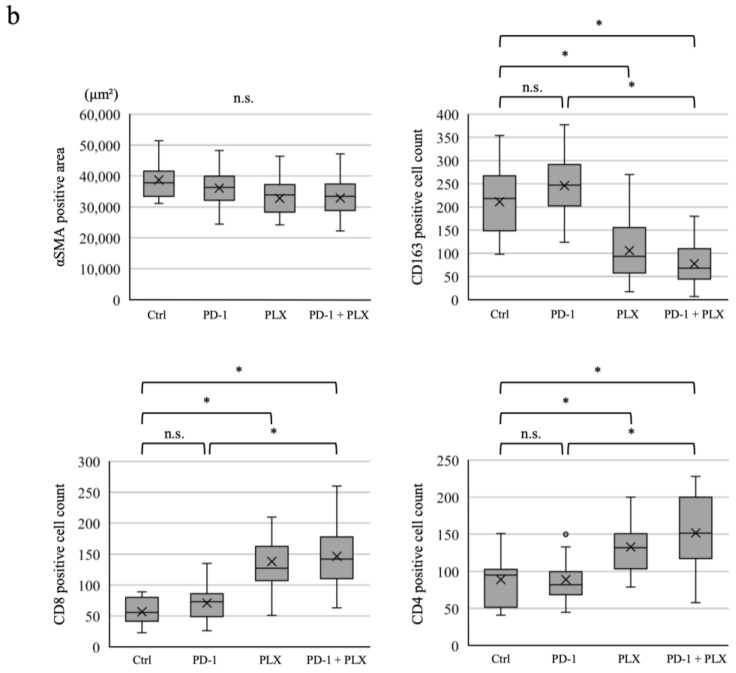
M2 macrophages induce an immunosuppressive orientation of the tumor microenvironment in an orthotopic mouse model of colorectal cancer (CRC). (**a**) Fluorescence immunostaining for αSMA, CD163, CD8, and CD4. (**b**) αSMA-positive areas and numbers of CD163-, CD8-, and CD4-positive cells in transplanted tumors in therapeutic experiments. Data are presented as means ± s.d.; * *p* < 0.01; n.s., not significant using an unpaired, two-sided Student’s *t*-test.

Mice were co-transplanted with MC38 and CAFs and divided into control, anti-PD-1 antibody monotherapy, PLX3397 monotherapy, and combination therapy groups, then dosed and sacrificed on day 35. No significant difference was noted between the control, anti-PD-1 antibody monotherapy, and PLX3397 monotherapy groups, but a significant reduction in tumor volume was observed with combination therapy (Figure 4b). Immunohistology of the resected transplanted tumors showed that CD8-positive T cells accumulated in the peritumoral stroma of tumor nests (red arrows), with little tumor infiltration in the control and anti-PD-1 antibody monotherapy groups, while those in the PLX3397 monotherapy and combination therapy groups showed significant tumor infiltration (Figure 4c). Ki67 labeling showed no change in the anti-PD-1 antibody and PLX3397 monotherapy groups, but was significantly decreased by combination therapy compared to controls (Figure 4c,d). The αSMA-positive area did not change significantly with either treatment (Figure 5a,b). The abundance of CD163-positive cells showed a decrease with PLX3397 monotherapy and combination therapy compared with controls, and no change with anti-PD-1 antibody monotherapy (Figure 5a,b). CD8-positive T cell infiltration was significantly increased by PLX3397 monotherapy and combination therapy, while no change was observed with anti-PD-1 antibody monotherapy compared to controls (Figure 5a,b). CD4-positive T cells also showed similar changes to those observed for CD8-positive T cells (Figure 5a,b). These results suggest that anti-PD-1 antibody monotherapy does not alter the immune composition of the tumor microenvironment, but depletion of M2 macrophages following PLX3397 administration induced infiltration of CD8-positive T cells into the tumor. The αSMA-positive area was not significantly changed by PLX3397, suggesting that the increased infiltration of CD8-positive T cells into the tumor was not due to a decrease in stroma, but due to a decrease in M2 macrophages.

### 2.5. Immune Pathways Are Activated in Transplanted Tumors When Anti-PD-1 Antibodies Are Combined with PLX3397

**Figure 6:**
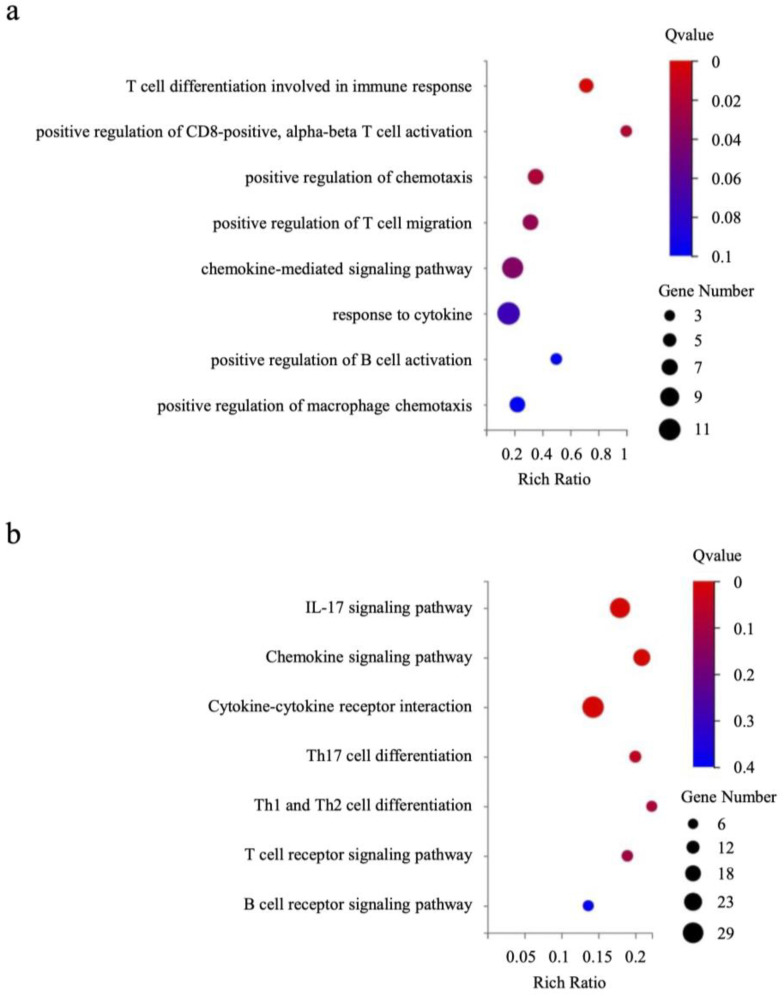
Gene expression analysis confirming immune pathway activation in transplanted tumors upon combined PLX3397 and anti-PD-1 antibody treatment. (**a**) Gene Ontology (GO) cellular component enrichment bubble chart. (**b**) KEGG pathway enrichment bubble chart.

In animal studies, a significant decrease in tumor volume was observed by combining anti-PD-1 antibody with PLX3397. We next extracted RNA from transplanted tumors from the four therapy groups and performed RNA-seq. Both Gene Ontology (GO) and Kyoto Encyclopedia of Genes and Genomes (KEGG) pathway analyses showed that immune gene expression pathways, primarily T cell-related, were significantly more activated by combination therapy than by anti-PD-1 antibody monotherapy (Figure 6a,b). That is, under conditions in which macrophage depletion allows CD8-positive T cells to infiltrate from the stroma into the tumor nest, immune cells activated by PD-1 blockade successfully bound to tumor cells and inhibited tumor cell growth.

## 3. Discussion

Newer cancer immunotherapies, such as immune checkpoint inhibitors, have been shown to be effective for treating multiple cancer types, with high expectations attached to their curative potential. However, there have been numerous cases for which adequate therapeutic efficacy has not been achieved [23,24]. In microsatellite instability CRC, the efficacy of particular immune checkpoint inhibitors has been demonstrated [2,3]. However, response rates remain low, and studies are underway to exploit T cell activation for more potent antitumor effects. In particular, the interaction between cancer cells, stromal cells, and immune cells in the tumor microenvironment, the frontline of the immune response, remains unclear.

Here, the expression level of CD206 in J774.1 cells 24 h after co-culture with CAFs was significantly higher than that with IL-4 stimulation, indicating that CAFs have the capability to strongly induce monocyte differentiation into M2 macrophages. This indicates that more M2 macrophages were present when CAFs were abundant (Figure 2b), further suggesting that CAFs recruit monocytes and induce their differentiation into M2 macrophages in human CRC as well. Cho et al. reported that, in vitro, CAFs derived from oral squamous cell carcinoma differentiated monocytes into M2 macrophages via secretion of IL-6 and GM-CSF upon stimulation by cancer cells [9]. Although we were unable to examine this in the present study, we speculate that monocytes are similarly induced to differentiate into M2 macrophages by the humoral factors produced by CAFs in CRC.

In co-cultures of J774.1 cells and MSCs, MSCs were significantly less able to induce monocyte differentiation than CAFs, and the phenomenon of attracting monocytes was not observed. MSCs are the precursors of CAFs. Although there are reports that MSCs promote the polarization of macrophages towards the M2 phenotype via CXCL12 and exosomal functions [25,26], there are no reports comparing the ability of MSCs and CAFs to induce differentiation. We have reported that MSCs differentiate into CAFs upon interaction with components of the tumor microenvironment [27], and our current investigation suggests that this differentiation may enhance their capability to induce differentiation of monocytes into TAMs and to promote monocyte migration.

We observed that CAFs promoted the migration of monocytes into the CRC tumor immune microenvironment, both in clinical specimens and in a murine orthotopic transplant model. Chemokines and cytokines produced by tumor and stromal cells, such as CAFs, induce monocytes in the peripheral blood to migrate to tissues and differentiate into TAMs [28]. CSF-1 is a major factor in this process [18], and other factors such as chemokine ligand (CCL)2 (MCP-1), CXC motif chemokine ligand 12 (SDF1α), vascular endothelial growth factor (VEGF), CCL20, and others have been reported to promote monocyte migration via their respective receptors on monocytes [29,30,31,32,33]. Li et al. reported that CAF effectively induced monocyte migration via the CXCL12/CXCR4 pathway, resulting in monocyte differentiation into TAMs in oral squamous cell carcinoma in vitro [10]. Zhang et al. reported that for CRC, IL-8 produced by CAFs induced monocyte migration via the IL-8/CXCR2 pathway and consequent polarization to M2 macrophages using cell motility assays [8]. However, to our knowledge, to date, no studies have examined the induction of monocyte migration and polarization to M2 macrophages by CAFs derived from CRC in multiple experimental systems in vitro, human clinical specimens, and in vivo. Cytokines produced by CAFs, including IL-6, VEGF, IL-8, HGF, and SDF-1, have been identified [34,35,36]; therefore, we hypothesized that in CRC, the humoral factors produced by CAFs also promoted the migration of monocytes into the tumor. This study did not include fibroblasts other than CAFs as a comparison; therefore, the effect of fibroblasts on monocytes or M2 macrophages may not be limited to CAFs.

We then investigated the effect of PLX3397 (Pexidartinib), a CSF-1R inhibitor targeting TAMs, on CRC using an orthotopic transplant mouse model of CRC. It has long been recognized that CRC contains abundant stroma [37], and we have previously reported that co-transplantation of stromal cells (such as MSCs and CAFs) with cancer cells in this orthotopic murine CRC model results in the formation of stroma-rich tumors [38,39]. To specifically investigate the behavior of CAFs and TAMs in the CRC microenvironment, we evaluated the effects of drugs on the tumor immune microenvironment using a stromal-rich allogeneic immunoreactive CRC mouse model, generated by orthotopic transfer of CRC cells and CAFs.

Although PLX3397 monotherapy did not significantly reduce tumor size in this study, it induced changes in immune composition, including a reduction in M2 macrophage abundance and infiltration of CD8-positive T cells into the tumor. Similar effects have been observed in studies on oral and breast cancers; CSF-1R inhibitor monotherapy resulted in depletion of M2 macrophages and an increase in CD8- and CD4-positive T cells, despite not suppressing tumor growth [21,40,41,42].

In this study, the combination of PLX3397 and anti-PD-1 antibody induced significant tumor shrinkage. Transcriptomics showed that T cell-related pathways were predominately activated in the combination therapy group. Emactuzumab (RG7155), an anti-CSF-1R antibody, has been reported to eliminate TAMs and increase T cell infiltration in a mouse model of CRC; further, its administration to humans depleted TAMs and increased the CD8/CD4 ratio [43], suggesting TAM suppression of T cell immunity. As a mechanism by which TAMs suppress T cell immune responses, IL-10, TGF-β, PGE2, and ARG1 have been reported as cytokines and other humoral factors [44,45,46,47]; moreover, the expression of PD-L1, an immune checkpoint molecule, is also reportedly involved [48]. A study in a breast cancer model also found that in the stroma, TAMs are in direct contact with CD8-positive T cells, reducing their motility and preventing them from infiltrating the tumor [21]. In our orthotopic transplanted tumors, numerous CD8-positive T cells were observed in contact with M2 macrophages, suggesting a similar interaction. It has also been reported that CD8-positive T cells in the tumor nest exhibit high expression of PD-1 after treatment with PLX3397 [21], suggesting that tumor immunity had been activated mainly in the T cell lineage by the combined use of anti-PD-1 antibody through a similar mechanism in the present study.

A dose-escalation phase I study is currently underway to evaluate the safety and activity of an anti-PDL1 antibody (Durvalumab) in combination with a small-molecule CSF-1R tyrosine kinase inhibitor (Pexidartinib) in patients with metastatic/advanced pancreatic cancer or CRC [49]. In tumors with abundant stromal components, such as pancreatic cancer and CRC, combining CSF-1R inhibitors with immunotherapy may be effective. However, further investigation is needed to identify the specific patient characteristics suitable for this treatment approach.

In conclusion, CAFs derived from CRC were found to induce differentiation of monocytes into M2 macrophages and promote monocyte migration into the tumor. In a murine model of CRC orthotopic transplantation, treatment with PLX3397 depleted M2 macrophages in the tumor and induced infiltration of CD8-positive T cells into the tumor, thereby enhancing the efficacy of immunotherapy with anti-PD-1 antibody. This combination therapy presents a new potential strategy for patients with CRC who do not respond adequately to immunotherapy alone.

## 4. Materials and Methods

### 4.1. Human CRC Samples

Overall, tumors excised from 73 patients diagnosed with CRC at Hiroshima University Hospital (Hiroshima, Japan) between 2013 and 2014 were collected. This study was conducted in accordance with the Declaration of Helsinki and was approved by the Institutional Review Board of Hiroshima University Hospital (approval number E2023-0171). Since the anonymized data and paraffin-embedded tissues used were from previously surgically resected samples, the Institutional Review Board waived the requirement for informed consent from each patient.

### 4.2. CAF and M2 Macrophage Immunohistology

To examine the correlation between the amount of CAFs and the number of M2 macrophages in human CRC tissue, 73 surgically resected tumor specimens were fixed in formalin, embedded in paraffin, and serially sectioned at a thickness of 4 μm. Fluorescence immunostaining was performed using anti-CD163 and anti-αSMA antibodies. Observations were made using a BZX710 fluorescence microscope (Keyence, Osaka, Japan). For each specimen, the microscopic field containing foci was photographed at 200× magnification and analyzed. The numbers of CD163 positive cells and αSMA-positive areas were measured by organizing and quantifying the luminance threshold using a hybrid cell-counting application (BZ-X analysis software, version 1.3.1.1; Keyence, Osaka, Japan).

### 4.3. Quantitative RT-PCR

Total RNA was extracted from mouse monocyte-like cell lines using the RNeasy Mini Kit (Qiagen, Hilden, Germany) according to the manufacturer’s instructions. Complementary DNA was synthesized from 1 μg of total RNA using the First-Strand cDNA Synthesis Kit (Amersham Biosciences, Piscataway, NJ, USA). After reverse transcription, qRT-PCR was performed using the LightCycler FastStart DNA Master SYBR Green I Kit (Roche Diagnostics, Basel, Switzerland) following the manufacturer’s recommended protocol. To correct for differences in RNA quality and quantity between samples, expression values were normalized to glyceraldehyde-3-phosphate dehydrogenase (GAPDH) values. Reactions were performed in triplicate. Primer sequences used for mRNA amplification of mouse GAPDH and mouse CD206 are shown in Table 2. Relative expression levels of CD206 in each cell line were calculated using the ΔΔC_T_ method with the murine monocyte-like cell line before stimulation as control.

### 4.4. Cell Lines

The BALB/c mouse colon cancer cell line CT26 (ATCC CRL-2639) was obtained from the American Type Culture Collection (Manassas, VA, USA). BALB/c mouse-derived MSCs were obtained from Cyagen Biosciences Inc. (Tokyo, Japan). The C57BL/6 mouse colon cancer cell line MC38 was obtained from the ALSTEM CELL ADVANCEMENTS (Richmond, CA, USA). C57BL/6 MSCs and the BALB/c monocyte-like cell line J774.1 were obtained from the RIKEN BioResource Research Center (Ibaraki, Japan).

### 4.5. Cell Culture and CAF Preparation

Cells (CT26, MC38, J774.1, and MSCs) were maintained in Dulbecco’s modified Eagle’s medium supplemented with 10% fetal bovine serum and penicillin–streptomycin mixture. CAFs were isolated from subcutaneously implanted tumors in mice as described in a previous report [50]. Isolated cells were sorted using fluorescence-activated cell sorting (FACS) to yield CD45-EpCAM-PDGFR+ cells, used as CAFs.

### 4.6. Co-Cultures

Six-well plates were seeded with J774.1 cells (3 × 10⁵ per well) as untreated negative controls. Plates seeded identically were treated with IL-4 (100 ng/mL), co-cultured with equal numbers of CAFs, or co-cultured with equal numbers of MSCs. J774.1 cells had been transfected with a fluorescent luciferase construct. After co-culture, J774.1 cells were sorted using flow cytometry, and the expression levels of each marker were analyzed by qRT-PCR. All experiments were performed in quadruplicate.

### 4.7. Animals

Female C57BL/6 mice were obtained from The Jackson Laboratory Japan, Inc. (Kanagawa, Japan). Animal experiments were approved by the Committee on Animal Experimentation at Hiroshima University (approval number A22-167). To produce cecal tumors, MC38 cells alone (1.0 × 10^6^) or MC38 cells mixed with CAFs in a ratio of 2:1 (1.0 × 10^6^:0.5 × 10^6^ MC38:CAFs) in 25 µL of Hanks’ balanced salt solution were injected into the cecal walls of C57BL/6 mice under a dissecting microscope. Five weeks after intracecal transplantation of these cells, the surviving mice were euthanized.

### 4.8. Murine CRC Model Immunohistochemistry

Formalin-fixed paraffin-embedded tumor tissues were serially sectioned at 4 µm thickness and immunostained with anti-CD163, anti-α-SMA, anti-CD8, anti-CD4, and anti-Ki67 antibodies. For fluorescence immunostaining, the OPAL 4-Color Manual IHC Kit (Perkin Elmer, Norwalk, CT, USA) was used. Observations were made using a BZX710 fluorescence microscope (Keyence, Osaka, Japan). For each specimen, the microscopic field containing the lesion was photographed at 200× or 400× magnification and analyzed. Positive regions for each antibody were measured by organizing and quantifying their luminance thresholds using a hybrid cell-counting application (BZ-X analysis software, version 1.3.1.1; Keyence, Osaka, Japan).

### 4.9. PLX3397 Treatment

Experiments were conducted using co-transplantation of cancer cells and CAFs. Mice were divided into four groups: a control group, receiving oral methylcellulose daily; a treatment group in which 20 mg/kg/day of anti-PD-1 antibody was injected intraperitoneally every 4 days; a treatment group given 30 mg/kg/day of oral PLX3397, a CSF-1R inhibitor, daily; and a treatment group that received both anti-PD-1 antibody and PLX3397 at the same doses. Drug administration was started 14 days after tumor transplantation; 35 days after transplantation, mice were euthanized, tumors were removed, and tumor diameters were measured. The tumor volume V was calculated as follows:

$$\left(V=\frac{\left(W^{2}\times L\right)}{2}\right)$$

where W is the short diameter and L is the long diameter of the tumor.

### 4.10. RNA Sequencing and Gene Set Enrichment Analysis (GSEA)

Orthotopically implanted tumors treated with anti-PD-1 antibody monotherapy or combination therapy with PLX3397 were dissected and mechanically disrupted using a homogenizer. Total RNA was extracted from homogenates using the RNeasy Mini Kit (Qiagen, Hilden, Germany) according to the manufacturer’s protocol. Library construction and data processing were performed at the Beijing Genome Institute (Beijing, China). The library was sequenced using the DNBSEQG400RS platform and high-quality reads were obtained. Sequence alignment was conducted using the GRCm38 mouse reference genome version GCF_000001635.26_GRCm38.p6 (https://www.ncbi.nlm.nih.gov/assembly/GCF_000001635.26, accessed on 10 March 2023)). Dr. Tom multiple omics data mining system (https://biosys.bgi.com, accessed on 18 December 2023; Beijing Genome Institute, Beijing, China) was used to identify relevant differentially expressed genes and perform enrichment analysis of GO and KEGG pathways.

### 4.11. Reagents

CSF-1R and c-Kit inhibitors along with pexidartinib (PLX-3397) were purchased from MedChemExpress (Monmouth Junction, NJ, USA). Anti-mouse PD-1 antibody, InVivoMAb (CD279), was purchased from Bio X Cell (Lebanon, NH, USA). Recombinant murine IL-4 was purchased from PeproTech (Cranbury, NJ, USA). Primary antibodies were anti-CD163 (NCL-L-CD163, Leica Biosystems, Nussloch, Germany); anti-CD163 (ab182422, Abcam, Cambridge, MA, USA); anti-CD4 rabbit mAb (D7D2Z, 25229S, Cell Signaling Technology, Danvers, MA, USA); anti-CD8α rabbit mAb (D4W2Z, 98941T, Cell Signaling Technology); anti-αSMA (ab5694, Abcam); and Ki-67 equivalent (GTX16667, GeneTex, Irvine, CA, USA). Antibodies used for FACS included PE anti-mouse CD326 (epithelial cellular adhesion molecule EpCAM; clone G8.8; BioLegend, San Diego, CA, USA), PE anti-mouse CD140a (PDGFR-α; clone G8.8; BioLegend), PerCP/Cyanine5.5 anti-mouse CD45 (clone 30-F11; BioLegend), and PE anti-mouse F4/80 (clone BM8; BioLegend).

### 4.12. Statistical Analysis

Clinicopathologic characteristics were analyzed using the χ^2^ test or Fisher’s exact test for comparing categorical data and Student’s *t*-test or the Mann–Whitney test for comparing continuous data. The Pearson product-moment correlation coefficient was used to index correlations. Kaplan–Meier curves and overall survival were analyzed using the log-rank test. Statistical significance was set at *p* < 0.05. All statistical analyses were performed using EZR software (version 1.60; Saitama Medical Centre, Jichi Medical University, Saitama, Japan).

## Figures and Tables

**Table 1:** Clinical characteristics of human CRC specimens.

**Number of patients**		73
**Age (years old)**		70.3 ± 9.5
Sex	Male	39 (53.4)
Location	Right side colon	26 (35.6)
	Left side colon	47 (64.4)
Histological Type	tub 1/2	65 (89.0)
	Por/muc	8 (11.0)
Stage	I/II	39 (53.4)
	III/IV	34 (46.6)
T	1/2	18 (24.7)
	3/4	55 (75.3)
N	N0	41 (56.2)
	N1/2/3	32 (43.8)
M	0	60 (82.2)
	1	13 (17.8)
Vascular invasion	0/1	63 (86.3)
	2/3	10 (13.7)
Budding grade	1	12 (48.0)
	2, 3	13 (52.0)
Microsatellite instability	MSS	66 (90.4)
	MSI-high	7 (9.6)

Data are represented as n (%). Abbreviations: tub, tubular adenocarcinoma; por, poorly differentiated adenocarcinoma; muc, mucinous adenocarcinoma; MSS, microsatellite stable; MSI-high, high frequency of microsatellite instability.

**Table 2:** qRT-PCR primers.

Target Gene	Direction	Sequence (5′-3′)
mouse GAPDH	Forward	GCCTCGTCCCGTAGACAAAA
	Reverse	CCATTCTCGGCCTTGACTGT
mouse CD206	Forward	GGAAACGGGAGAACCATCAC
	Reverse	GGCGAGCATCAAGAGTAAAG
mouse iNos	Forward	AGGGACAAGCCTACCCCTC
	Reverse	CTCCATCTCCCGTCAGTTGGT
mouse IL-1	Forward	TCACAGCAGCACATCAACAA
	Reverse	TGTCCTCATCCTGGAAGGT
mouse IL-6	Forward	GTCCTTCAGAGAGATACAGAAACT
	Reverse	AGCTTATCTGTTAGGAGACCATTG

## Data Availability

The data that support the findings of this study are available from the corresponding author upon reasonable request.
